# The Potential and Prospects of Hydrogel Applications in Traumatic Brain Injury Treatment

**DOI:** 10.3390/cimb48050488

**Published:** 2026-05-08

**Authors:** Cheng Zhong, Jie Li, Dengzhuo Liu, Xinran He, Zihao Fan, Xinxin Guo, Guangwei Wang

**Affiliations:** 1 School of Clinical Medicine, Youjiang Medical University for Nationalities, Baise 533000, China; 2 School of Medical Information and Engineering, Hunan University of Medicine, Huaihua 418000, China; 3 School of Basic Medical Sciences, Hunan University of Medicine, Huaihua 418000, China; 4 Graduate School, University of South China, Hengyang 421200, China

**Keywords:** hydrogel, traumatic brain injury, neural regeneration, inflammation regulation, drug delivery

## Abstract

Traumatic brain injury (TBI) is a prevalent neurological disorder that induces severe neurological dysfunction and markedly reduces quality of life owing to its complex pathophysiology and limited therapeutic options. Conventional pharmacological and surgical interventions show restricted efficacy because of poor blood–brain barrier penetration and inability to address secondary injury cascades. In recent years, hydrogels have shown significant potential for TBI repair due to their superior biocompatibility, high water content, and ability to mimic the native extracellular matrix (ECM). This review systematically examines recent advances in hydrogel applications for TBI therapy, focusing on their roles as drug delivery platforms, stem cell scaffolds, neuroregeneration promoters, inflammation modulators, and angiogenesis facilitators. Particular emphasis is placed on the therapeutic benefits and underlying mechanisms of ECM-derived hydrogels, self-assembling peptide (SAP) hydrogels, stimuli-responsive smart hydrogels, and functionalized multicomponent systems. Current challenges and limitations in hydrogel applications are also discussed, along with future research directions, to provide scientific rationale and practical guidance for precision TBI therapy.

## 1. Introduction

Traumatic brain injury (TBI) represents a major global public health concern, characterized by high rates of neurological impairment, disability, and mortality. It imposes profound physical and cognitive deficits on patients, placing considerable economic and psychological burdens on families and society. The pathophysiology of TBI is complex, involving primary mechanical injury followed by secondary injury cascades at the molecular, metabolic, and cellular levels. Secondary brain injury encompasses various pathophysiological processes, including blood–brain barrier (BBB) disruption, neuroinflammation, oxidative stress, apoptosis, and neurodegeneration. These cascades progressively exacerbate brain tissue damage and impede neurological recovery [1,2,3].

Current TBI treatments primarily rely on pharmacological and surgical interventions. However, the BBB and its selective permeability severely restrict the delivery of many systemically administered drugs to brain tissue. This results in several limitations, including poor targeting specificity, difficulty in maintaining therapeutic drug concentrations, and overall suboptimal efficacy. Moreover, while surgical interventions can alleviate localized compression, they do little to halt the progression of secondary injury mechanisms [4,5,6]. Consequently, there is an urgent need to develop innovative therapeutic strategies that can overcome BBB limitations, precisely target injured brain tissue, mitigate secondary injury, and promote neuroprotection and regeneration.

In recent years, hydrogels—three-dimensional polymeric networks with high water content, excellent biocompatibility, and tunable physicochemical properties—have garnered significant attention for brain injury repair owing to their structural similarity to the native brain extracellular matrix (ECM). These hydrogels not only fill tissue defects and provide structural support but also enable the encapsulation and controlled release of drugs, growth factors, stem cells, and nanocarriers, facilitating precise localized delivery. Furthermore, by fine-tuning their mechanical properties, degradation kinetics, and bioactivity, hydrogels can adapt to the dynamic brain microenvironment, thereby promoting neuronal survival, axonal regeneration, inflammation modulation, and angiogenesis [7,8]. This review provides a systematic overview of recent advances in hydrogel-based therapies for TBI, with particular emphasis on their mechanisms in neuroprotection, regulation of neuroinflammation, neuroregeneration, and angiogenesis. By examining hydrogel classifications, their carrier functionalities, and their integration with drug and cell therapies, we highlight their potential to overcome the limitations of conventional treatments. Ultimately, this review aims to offer novel insights and a theoretical foundation for the clinical management of TBI, accelerate the translational application of hydrogel technologies, improve patient outcomes, and reduce the societal burden of this condition.

## 2. Literature Search Strategy and Selection Criteria

We conducted a systematic literature search of PubMed, Web of Science, Scopus, and Google Scholar using the combined keywords (“hydrogel” OR “hydrogels”) AND (“traumatic brain injury” OR TBI OR “brain trauma”). The search covered publications from January 2015 to December 2025. Inclusion criteria were as follows: (1) original research articles or reviews reporting hydrogel applications (as drug delivery platforms, scaffolds, stem cell/exosome carriers, or stimuli-responsive systems) in preclinical TBI models or clinically relevant in vitro studies; (2) English-language peer-reviewed publications. Exclusion criteria included non-hydrogel biomaterials, conference abstracts, and non-English articles. Approximately 200 records were initially screened by title and abstract, followed by full-text assessment, ultimately resulting in the inclusion of more than 80 high-quality studies. This transparent and reproducible search strategy ensures that the selection process is comprehensive rather than ad hoc and allows readers to evaluate potential selection bias.

## 3. Pathological Mechanisms and Therapeutic Challenges in TBI

### 3.1. Primary and Secondary Injury Mechanisms in TBI

TBI is defined as damage to brain tissue caused by external mechanical forces. The pathophysiological process comprises two distinct phases: primary and secondary injury. Primary injury involves immediate mechanical disruption of brain tissue by external forces, including direct parenchymal damage, vascular injury, and neuronal death. These injuries occur instantaneously upon impact, are irreversible, and result in immediate structural and functional impairment of the brain. Examples include cerebral contusions, hemorrhages, and diffuse axonal injury [9]. The severity of primary injury correlates with the magnitude and location of the trauma and often dictates early clinical presentation and prognosis.

**Figure 1:**
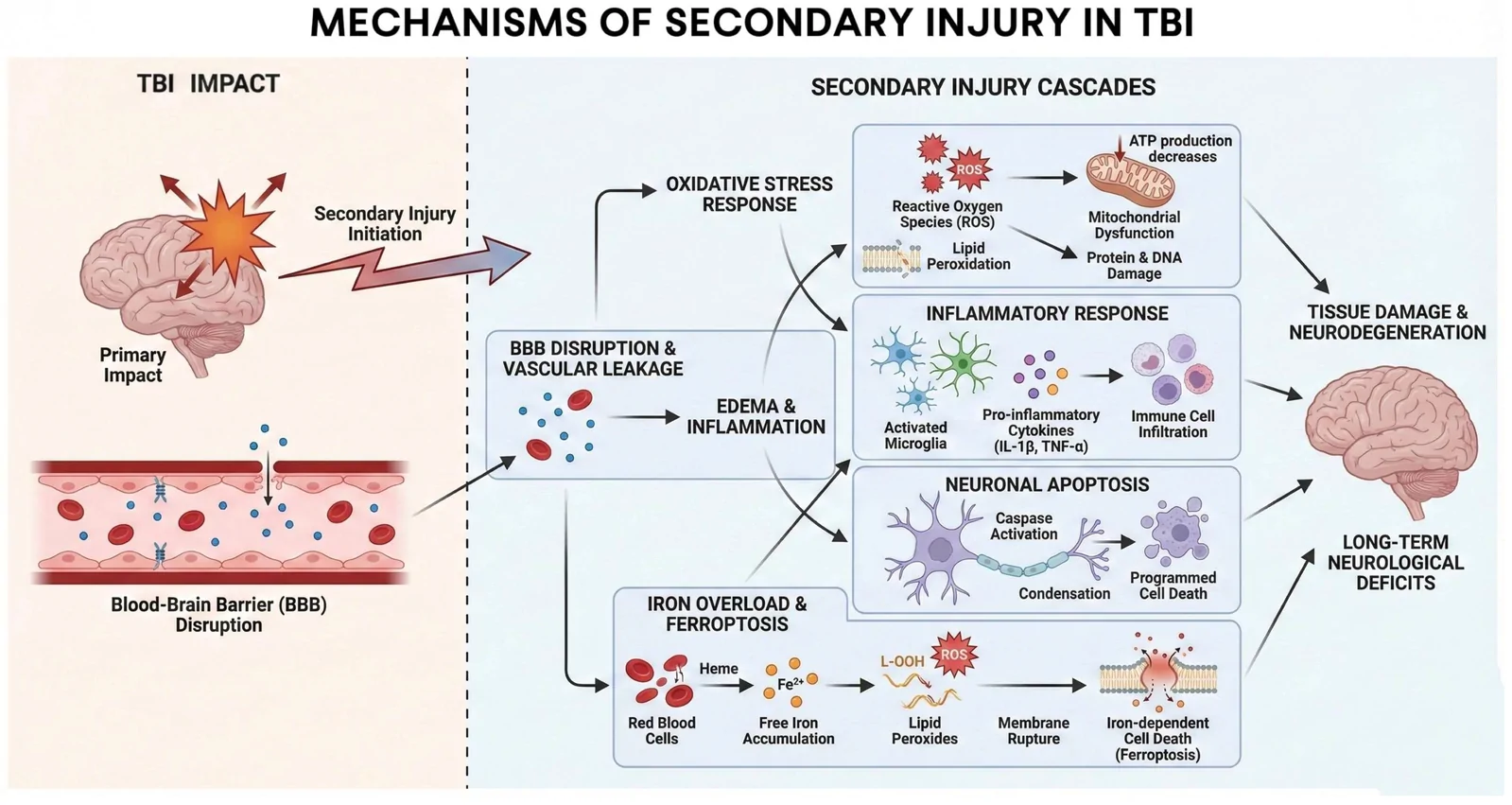
Secondary injury in TBI.

Secondary injury, in contrast, involves progressive brain tissue damage initiated by complex biochemical and cellular cascades triggered after the primary insult. This phase develops over hours to days after injury and encompasses multiple mechanisms, including BBB disruption, oxidative stress, inflammation, neuronal apoptosis, and iron overload-induced ferroptosis [9,10,11] (Figure 1). BBB disruption allows infiltration of blood components and inflammatory mediators into the brain parenchyma, thereby triggering an inflammatory cascade that exacerbates cerebral edema and necrosis [12]. Oxidative stress is characterized by excessive production of reactive oxygen species (ROS) and reactive nitrogen species (RNS), leading to lipid peroxidation, protein denaturation, and DNA damage [13]. Inflammation is primarily mediated by activated microglia and infiltrating peripheral immune cells that release pro-inflammatory cytokines, such as interleukin-1β (IL-1β) and tumor necrosis factor-α (TNF-α), thereby promoting neuronal apoptosis and further tissue damage [13,14]. In addition, iron overload induces severe lipid peroxidation, which not only directly damages the cell membrane—leading to ferroptosis—but also impairs mitochondrial function. Damaged mitochondria subsequently release mitochondrial DNA and excessive mitochondrial reactive oxygen species, thereby promoting assembly and activation of the NOD-like receptor family pyrin domain containing 3 (NLRP3) inflammasome. Activated NLRP3 then triggers Caspase-1, which cleaves Gasdermin D, enabling pore formation in the cell membrane. This process causes cell swelling and rupture, the release of pro-inflammatory cytokines such as IL-1β and interleukin-18 (IL-18), and ultimately pyroptosis, further exacerbating brain tissue damage [15]. Persistent secondary injury is closely associated with progressive neurological dysfunction and the development of neurodegenerative disorders [16].

Studies have shown that early serum metabolic perturbations in patients with severe TBI reflect both primary injury-induced disruption of energy metabolism and dynamic changes in neurotransmitters and inflammatory mediators during secondary injury [9]. Moreover, animal models and in vitro studies demonstrate that TBI not only induces localized damage but also propagates to remote brain regions through enhanced neuronal apoptosis and inflammation, ultimately resulting in widespread neuropathological changes [13,16]. Clinically, targeting secondary injury processes—such as intracranial pressure management, inflammation suppression, and oxidative stress mitigation—is critical for improving patient outcomes [16,17].

In summary, primary injury in TBI involves irreversible mechanical disruption of brain parenchyma, vasculature, and neurons. Secondary injury, conversely, drives progressive and widespread brain damage through BBB disruption, oxidative stress, inflammatory cascades, apoptosis, and iron overload-induced ferroptosis (Figure 1). These secondary injury mechanisms constitute key therapeutic targets. A deeper understanding of these processes and their interactions will facilitate the development of effective interventions to mitigate long-term neurological sequelae in TBI patients [9,10,12].

Following TBI, the primary insult initiates secondary injury cascades: BBB disruption and vascular leakage drive edema and inflammation, accompanied by oxidative stress (ROS, lipid peroxidation, mitochondrial dysfunction), inflammatory activation (microglia, cytokines, immune infiltration), neuronal apoptosis, and iron overload-induced ferroptosis, culminating in tissue damage, neurodegeneration, and long-term neurological deficits.

### 3.2. Limitations of Conventional Treatments

**Figure 2:**
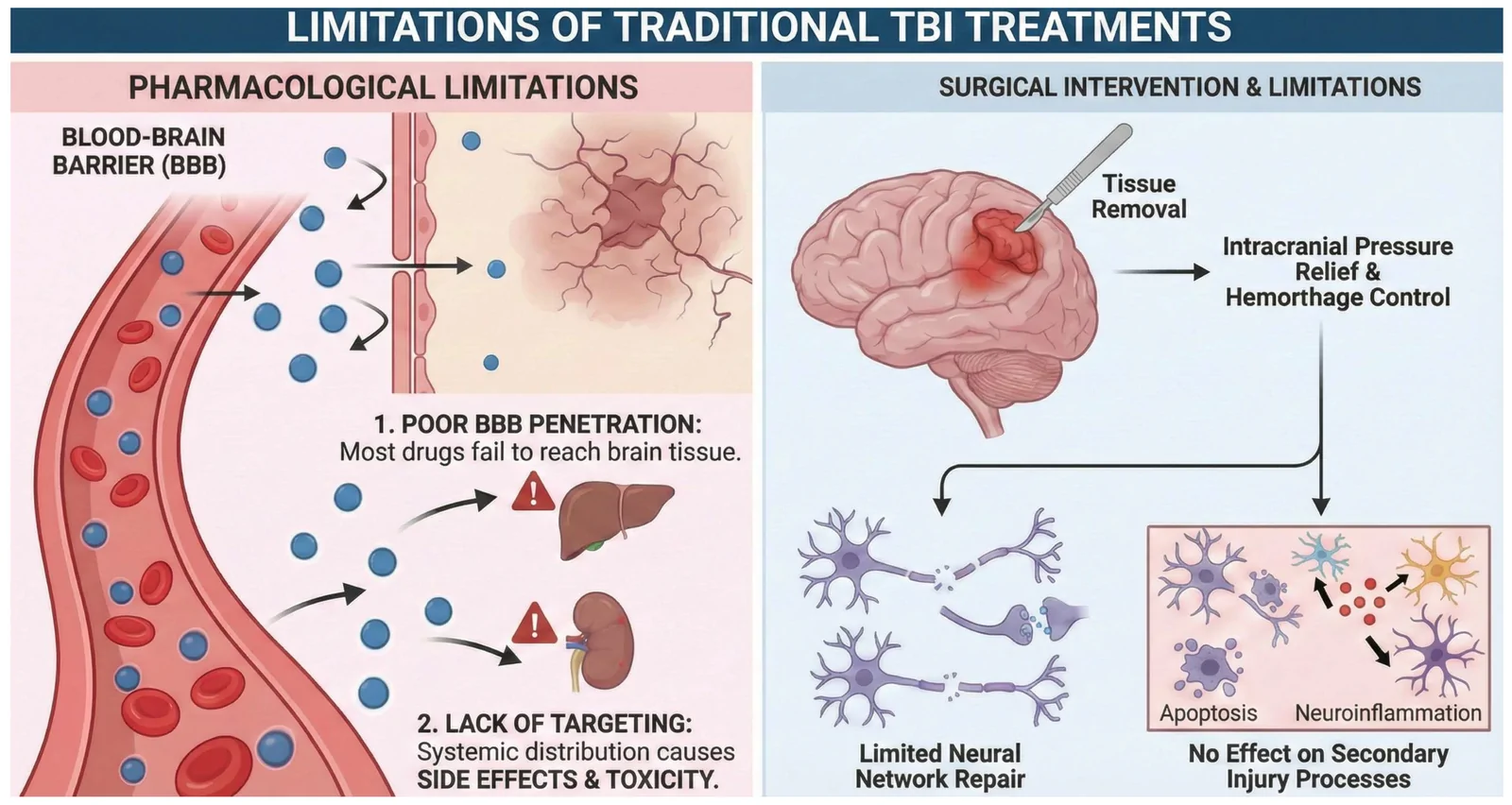
Limitations of conventional treatment for TBI.

Conventional TBI treatments primarily rely on pharmacological and surgical interventions. However, these approaches are limited by several inherent constraints that significantly compromise their therapeutic efficacy. Pharmacotherapy is particularly hindered by the BBB, which impedes the penetration of most drugs into brain tissue and thereby reduces therapeutic effectiveness (Figure 2). Although the BBB serves as a protective barrier against harmful substances, it simultaneously restricts effective drug delivery to the injured brain. Consequently, many neuroprotective and anti-inflammatory agents fail to reach therapeutic concentrations following systemic administration, resulting in diminished efficacy in TBI. Moreover, conventional pharmacotherapies often lack specificity, leading to off-target systemic side effects and toxicity that further restrict their clinical utility [18,19] (Figure 2).

Surgical interventions can effectively excise damaged tissue, alleviate intracranial pressure, and control hemorrhage; however, they offer limited capacity to reconstruct neural networks or restore neurological function (Figure 2). While surgery primarily addresses the structural deficits caused by primary mechanical injury, it is largely ineffective against the progressive secondary injury cascades, including neuronal apoptosis and neuroinflammation. As a result, patients frequently exhibit persistent motor and cognitive deficits postoperatively, with only limited functional recovery [20,21,22]. Furthermore, surgical procedures may themselves provoke additional secondary injury by inducing intensified inflammation and cerebral edema, thereby worsening prognosis.

Innovative materials and advanced technologies are urgently needed to overcome these limitations by improving drug delivery and facilitating brain tissue repair. For example, nanomedicine utilizes nanomaterials as carriers to enhance BBB penetration, thereby enabling targeted delivery and augmenting both neuroprotection and diagnostics [18,23]. Biomaterials such as hydrogels serve as local drug delivery platforms and tissue engineering scaffolds, facilitating sustained release at the injury site and promoting neural regeneration, thus overcoming the targeting limitations of conventional therapies. Additionally, stem cell therapy outperforms conventional approaches in tissue repair owing to its multidirectional differentiation potential, self-renewal capacity, and immunomodulatory properties. In particular, mesenchymal stem cells (MSCs) and their exosomes show considerable promise in modulating neuroinflammation and promoting neuroregeneration [24,25].

In summary, conventional therapies for TBI are limited by inadequate BBB penetration and insufficient neural network reconstruction, which together hinder functional recovery. Future strategies should harness emerging technologies—such as stimuli-responsive hydrogels, nanocarriers, and stem cell therapies—to achieve precise drug delivery and effective neural repair, ultimately improving therapeutic efficacy and patient outcomes [26].

Limitations of traditional TBI treatments: Pharmacotherapy is constrained by the BBB, leading to poor brain penetration and limited targeting with systemic toxicity/side effects; surgical interventions (tissue removal, hemorrhage control, intracranial pressure relief) provide acute stabilization but offer limited neural network repair and have minimal impact on secondary injury processes (e.g., apoptosis and neuroinflammation).

## 4. Material Properties of Hydrogels and Their Advantages in TBI

### 4.1. Physicochemical Properties of Hydrogels

**Figure 3:**
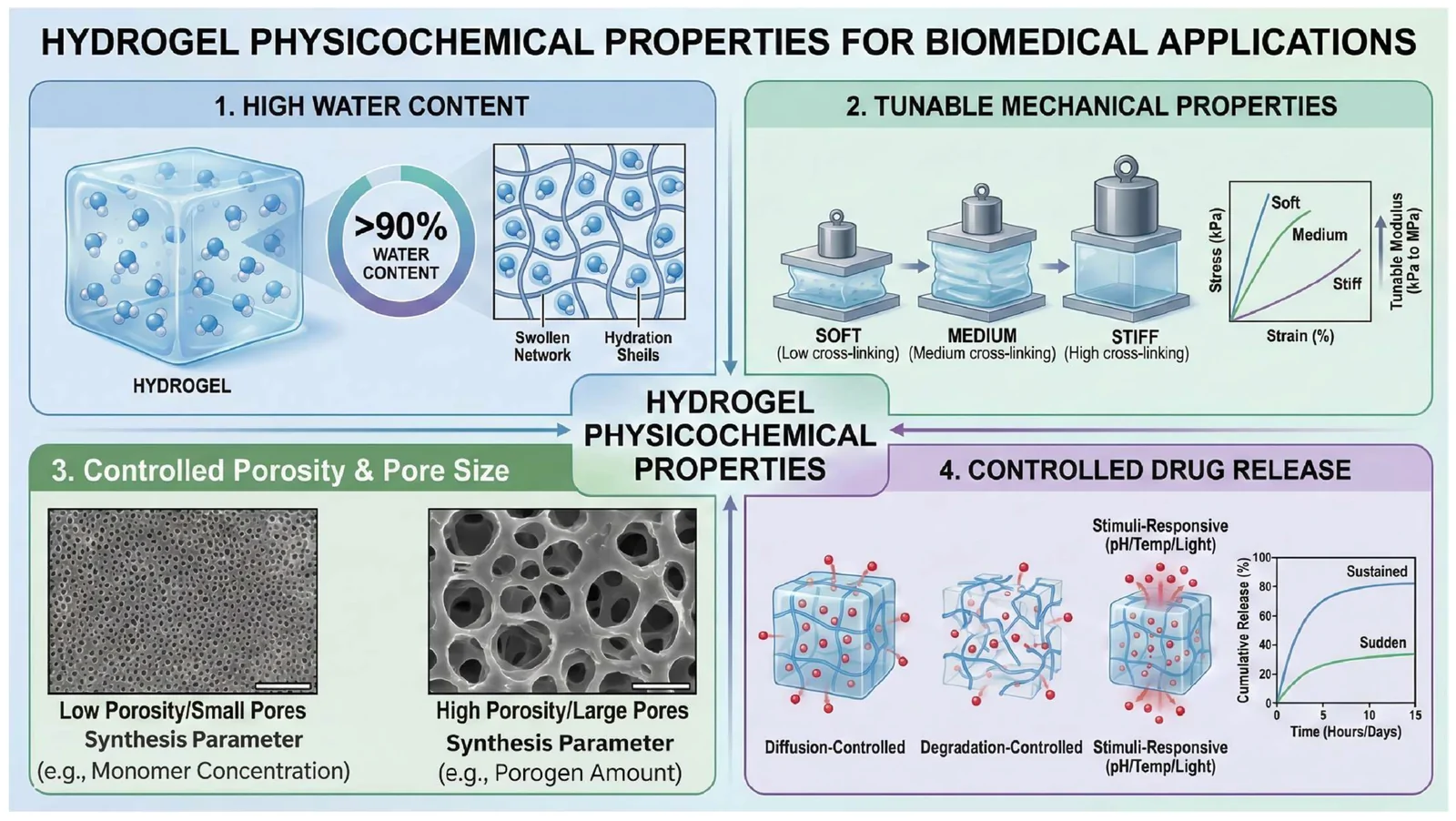
Physicochemical properties of hydrogels.

Hydrogels are three-dimensional hydrophilic polymer networks characterized by exceptionally high water content, which confers distinct advantages for TBI therapy (Figure 3). This high water content not only imparts a soft texture comparable to native biological tissues but also establishes a hydrated microenvironment that closely mimics the ECM, thereby promoting cell adhesion, proliferation, and differentiation. For example, carboxymethyl cellulose (CMC)-based hydrogels, renowned for their excellent biocompatibility and superior water absorption capacity, are particularly suitable for biomedical applications. Studies have shown that carboxyl-crosslinked CMC hydrogels exhibit markedly enhanced swelling ratios in both water and phosphate-buffered saline (PBS), along with favorable thermal stability and mechanical strength, thereby meeting the essential requirements for tissue regeneration scaffolds [27].

Furthermore, the mechanical properties of hydrogels are highly tunable. By modulating crosslinker type, density, polymer concentration, and composition, their elastic moduli can be precisely matched to those of brain tissue (Figure 3). For instance, electrostatically crosslinked carrageenan/chitosan hydrogels exhibit pH-dependent changes in storage modulus and swelling ratio, enabling adaptation to diverse physiological environments and providing a versatile platform for drug delivery and tissue engineering [28]. Similarly, crosslinking collagen hydrogels with natural polyphenols such as gallic acid or ellagic acid significantly enhances storage modulus, thermal stability, and resistance to enzymatic degradation. The resulting hydrogen bonding and hydrophobic interactions produce denser networks with superior mechanical properties, thereby expanding their biomedical applicability [29]. At the microstructural level, hydrogels closely replicate the brain ECM milieu. Their porous three-dimensional architecture, with pore size and porosity readily tuned through fabrication parameters, offers abundant adhesion sites and sufficient space for cellular ingrowth. For example, CMC–starch hydrogels processed via thermal and physical methods form uniform porous structures that promote cell infiltration and efficient nutrient exchange [30] (Figure 3). Moreover, natural polysaccharide hydrogels—including alginates, chitosan, and hyaluronic acid—offer excellent biocompatibility and tunable mechanical properties, effectively mimicking the brain ECM to support neural cell adhesion and regeneration [31,32,33]. These physicochemical properties can be simultaneously optimized without compromising biocompatibility by employing modular crosslinking strategies, precise control of polymer concentration, and strategic incorporation of nanoparticles. For instance, fine-tuning crosslinking density enables the storage modulus to be adjusted within the physiologically relevant range of 0.1–10 kPa while preserving water content above 90% and maintaining excellent cell viability, as demonstrated in multiple studies [34,35].

Hydrogels also possess stimuli-responsive and controlled-release capabilities. Through chemical modification and nanoparticle integration, they can respond to external stimuli such as temperature, pH, or magnetic fields, thereby dynamically modulating their mechanical properties and drug release kinetics (Figure 3). For example, thermosensitive chitosan/gelatin/glycerol phosphate hydrogels loaded with ferulic acid provide robust mechanical support and localized antioxidant activity in TBI models, making them highly suitable for managing oxidative stress after traumatic brain injury [36]. Likewise, nanocomposite hydrogels incorporating nanoclay and hydroxyapatite exhibit enhanced mechanical properties and bioactivity, thereby promoting cell proliferation and tissue regeneration [34,35,37].

Representative studies provide concrete quantitative evidence supporting these physicochemical advantages. For example, the ROS-responsive hydrogel composed of phenylboronic acid-modified hyaluronic acid (HA-PBA), polyvinyl alcohol (PVA), and deferoxamine (DFO), hereafter referred to as the HA-PBA/PVA/DFO hydrogel, displays a storage modulus of 0.5 kPa—closely matching the softness of native brain tissue—rapid gelation within 5 min, and controlled degradation over 7–14 days under physiologically relevant ROS levels (0.1–1 mM H_2_O_2_), accompanied by approximately 80% release of DFO during this period [38]. Similarly, ECM-derived hydrogels composed of hyaluronic acid (HA), gelatin (Gel), salvianolic acid B (SAB), and vascular endothelial growth factor (VEGF), hereafter referred to as HA/Gel/SAB/VEGF hydrogels, exhibit a tunable storage modulus ranging from 0.2 to 1.2 kPa, swelling ratios exceeding 90%, and sustained release of VEGF over 4–8 weeks, culminating in 40–60% reduction in lesion volume in C57BL/6 mice [39]. Gelatin methacryloyl (GelMA)-based stem cell-loaded platforms further demonstrate in vivo degradation rates of 4–12 weeks and elastic moduli of 0.1–0.8 kPa that support >70% neural stem cell viability and differentiation [40]. These precisely quantified parameters, obtained from representative TBI models, supplant earlier descriptive accounts with study-specific metrics, clearly illustrating how hydrogel properties can be rationally engineered for optimal performance in TBI repair [38,39,40].

In summary, the key physicochemical attributes of hydrogels—high water content, excellent biocompatibility, tunable mechanical properties, and microporous architecture—enable effective mimicry of the brain ECM, thereby supporting cell adhesion and proliferation. Moreover, precise engineering of their structure and function through strategic material selection, crosslinking strategies, and nanoparticle compositing provides an ideal platform for TBI repair [27,29,31,33].

Physicochemical properties of hydrogels for biomedical applications: high water content (often >90%) with a hydrated polymer network; tunable mechanical properties via crosslinking density (soft to stiff); controlled porosity and pore size (e.g., adjusted by porogens or monomer concentration) affecting transport and biointeraction; and controlled drug release through diffusion-controlled, degradation-controlled, or stimulus-responsive (pH, temperature, and light) mechanisms.

### 4.2. Smart Responsive Properties of Hydrogels

Stimuli-responsive hydrogels detect local environmental cues and undergo rapid physical or chemical changes under specific conditions, thereby enabling targeted drug release and promoting tissue repair. Recent advances in materials science and biomedicine have underscored the substantial potential of these hydrogels in TBI therapy, particularly for achieving precise spatiotemporal control of drug delivery and mimicking the dynamic physiological microenvironment [8,41].

Stimuli-responsive hydrogels dynamically modulate their physical properties and drug release kinetics in response to local microenvironmental cues, such as pH, temperature, and ROS. For instance, pH-responsive hydrogels detect the acidic environment at injury sites and undergo structural transitions that enable selective drug release, thereby enhancing accumulation at the lesion and minimizing systemic adverse effects [42]. Temperature-responsive hydrogels utilize physiological heat or localized thermal signals to induce reversible sol–gel transitions, thereby providing controlled drug release [43,44]. In addition, ROS-responsive hydrogels, designed to target oxidative stress at TBI sites, undergo structural degradation or relaxation at elevated ROS levels, facilitating intelligent, on-demand drug release to support tissue repair [41,42,45].

These mechanisms depend on dynamic crosslinks or reversible chemical bonds (e.g., ester, imine, and thioether linkages) that cleave or reform in response to stimuli, thereby altering hydrogel morphology and drug diffusion rates [8,46]. To further enhance intelligence and targeting precision, multi-stimuli-responsive hydrogels that integrate pH, temperature, and ROS triggers have been developed, providing versatile and adaptive control within the complex post-TBI microenvironment [8,41]. Additionally, certain designs incorporate optical or electrochemical sensors for real-time monitoring of local parameters (e.g., pH, temperature, and ROS levels), which facilitates precise drug delivery and objective efficacy assessment. Such sensor-integrated hydrogels hold considerable promise for broad TBI applications by improving both therapeutic efficiency and safety [46,47]. In particular, multi-stimuli-responsive systems have shown synergistic therapeutic effects in preclinical brain disease models. For example, poloxamer-based hydrogels that integrate pH-sensitive ionization, ROS-triggered bond cleavage, and thermosensitive sol–gel transition achieve 2–3-fold greater drug penetration and neuroprotection than single-stimulus controls. This superior performance stems from more precise spatiotemporal control of release kinetics, higher local drug concentrations at the injury site, reduced systemic side effects, and superior adaptation to the complex post-injury microenvironment characterized by acidic pH, elevated ROS levels, and physiological temperature fluctuations. Although direct evidence from TBI models is still limited and emerging, these findings underscore the considerable potential of triple-responsive hydrogels to simultaneously address multiple pathological cues in TBI, thereby paving the way for more intelligent and effective therapies [41,48].

Dynamic crosslinking is a hallmark of stimuli-responsive hydrogels. Non-covalent interactions (e.g., hydrogen bonds, van der Waals forces, and electrostatic interactions) or reversible covalent bonds enable rapid network reconfiguration, imparting self-healing ability, deformability, and mechanical stability [46,47,49]. These features confer flexibility and plasticity, allowing hydrogels to more closely mimic the mechanical behavior of native ECM. Consequently, they facilitate cell migration and proliferation, thereby promoting tissue regeneration [50,51]. In particular, hydrogels featuring reversible crosslinking structures can achieve adaptive mechanical matching, enhance cell migration and tissue integration, support controlled degradation, and enable intelligent, multifunctional responses in TBI repair.

Adaptive mechanical matching is realized through dynamic crosslinking, allowing the hydrogel to conform to the softness of brain tissue while retaining sufficient mechanical strength, thereby minimizing secondary damage from mechanical mismatch [46]. The dynamic network structure further promotes cell migration and tissue integration by permitting cell infiltration and migration while supporting extracellular matrix remodeling, ultimately facilitating neural cell growth and functional recovery [50]. Controlled and sustained degradation is achieved via reversible cleavage and reformation of crosslinks, enabling the hydrogel to degrade progressively in response to local microenvironmental changes. This approach prevents long-term material retention that could impair tissue function while simultaneously releasing growth factors or therapeutic agents to accelerate healing [45,51]. Smart responsiveness and multifunctional integration stem from the combination of dynamic structures and multi-stimulus sensitivity, enabling flexible adaptation of both structure and function within the TBI microenvironment for precise therapeutic delivery [46,52]. Furthermore, modulation of crosslink density and network topology in dynamic hydrogels provides spatiotemporal control of cellular behavior, thereby optimizing the physicochemical microenvironment for post-TBI neural regeneration and recovery [47,50,51].

In summary, stimuli-responsive hydrogels that integrate dynamic crosslinking and reversible structures offer a powerful new platform for TBI therapy. By sensing and responding to local microenvironmental changes, they enable precise targeted delivery and effective tissue repair while demonstrating substantial translational potential. Future research should focus on optimizing stimulus sensitivity, mechanical properties, and biocompatibility to accelerate clinical translation for TBI management [53].

### 4.3. Biological Functional Advantages of Hydrogels

Hydrogels are three-dimensional hydrated polymer networks that serve as ideal biomaterials for TBI therapy owing to their close structural resemblance to native brain tissue. Their principal biological advantages include promotion of neural stem cells (NSCs) adhesion, proliferation, and differentiation; facilitation of angiogenesis and optimization of the local microenvironment; and anti-inflammatory, antioxidant, and anti-apoptotic effects, all of which help attenuate secondary injury after TBI [54,55].

**Figure 4:**
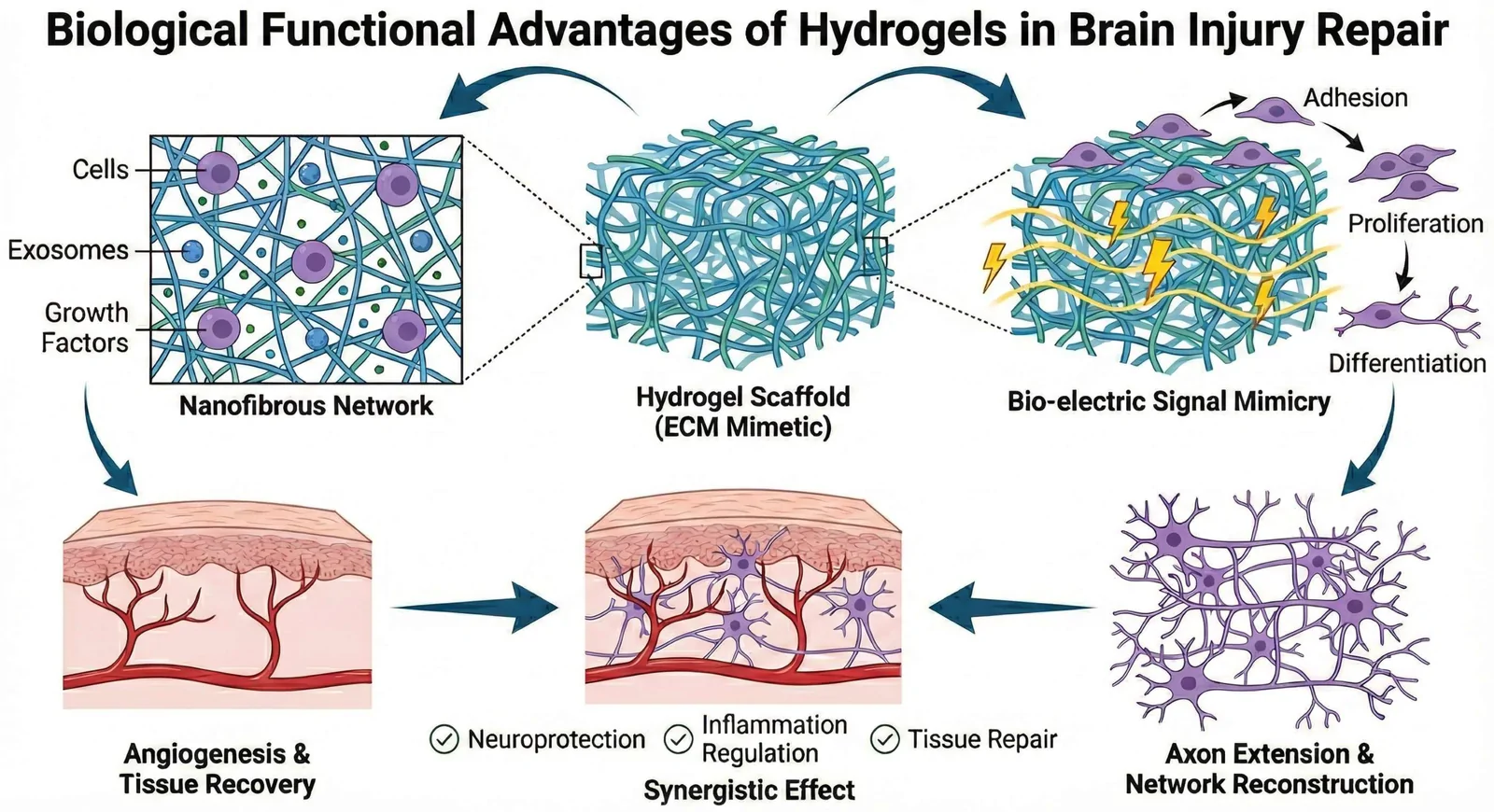
Biofunctional advantages of hydrogels.

Hydrogels closely mimic the three-dimensional ECM, thereby providing an optimal platform for NSCs adhesion and proliferation (Figure 4). Protein-based hydrogels (e.g., collagen, gelatin, and sericin) regulate stem cell behavior through bioactive motifs, thereby promoting adhesion, proliferation, and neural differentiation. For example, the co-delivery of sericin nanomaterials with NSCs markedly enhanced axon outgrowth and neurotrophic factor secretion, ultimately improving functional recovery in injured brain regions [56]. Moreover, functionalized collagen-like recombinant proteins incorporated into GelMA hydrogels improve cell viability and migration, thereby accelerating wound healing [40]. Collectively, these findings demonstrate that hydrogels not only supply structural support but also actively modulate the cellular microenvironment to enhance the regenerative capacity of NSCs.

Hydrogels further facilitate angiogenesis, thereby optimizing the microenvironment at the injury site (Figure 4). Angiogenesis plays a critical role in neural repair. These materials promote neovascularization and restoration of microcirculation through the encapsulation and controlled release of angiogenic factors (e.g., VEGF and basic fibroblast growth factor (bFGF)) or functionalized pro-angiogenic molecules. For example, an ECM-derived hydrogel engineered with bFGF enabled sustained release in a cerebral ischemia model, thereby preserving neuronal survival, promoting angiogenesis, and accelerating functional recovery [40]. Similarly, 3D-printed hydrogels incorporating platelet-rich plasma (PRP) and nanoclay enhanced angiogenesis and tissue regeneration while displaying robust bioactivity [57]. Multifunctional hydrogels further augment neural repair by stimulating vascular endothelial cell migration and differentiation [39]. By optimizing the local microenvironment, hydrogels create stable, nutrient-rich conditions that support neural cell survival and function.

Hydrogels also exhibit anti-inflammatory, antioxidant, and anti-apoptotic properties that help attenuate secondary injury after TBI (Figure 4). Following TBI, excessive inflammation and oxidative stress are major drivers of neuronal death and persistent functional deficits. Functionalized hydrogels, such as gallic acid-modified hyaluronic acid derivatives, effectively scavenge ROS, suppress pro-inflammatory cytokines, and promote neural repair [48]. Moreover, ROS-responsive hydrogels loaded with DFO alleviate iron overload and oxidative stress, thereby protecting neurons and facilitating functional recovery [38]. Similarly, hyaluronic acid/gelatin hydrogels incorporating crocin A and tannic acid A exert combined anti-inflammatory and pro-angiogenic effects, resulting in reduced lesion volume and improved neurological function [39]. Collectively, these hydrogels create a reparative microenvironment by modulating immune cell polarization, downregulating pro-inflammatory cytokines, and alleviating neuroinflammation.

In summary, hydrogels, with their biomimetic structures and tunable biofunctionality, promote NSCs adhesion, proliferation, and directed differentiation; support angiogenesis; and optimize the injured brain microenvironment. In addition, they exert potent anti-inflammatory, antioxidant, and anti-apoptotic effects that markedly attenuate secondary injury after TBI. Collectively, these attributes highlight the substantial therapeutic potential of hydrogels in TBI management [38,39,48,56,58].

Biofunctional advantages of hydrogels for brain injury repair: they mimic the extracellular matrix and provide a 3D scaffold to support neural stem cell adhesion, growth, and proliferation; promote angiogenesis and modulate the local microenvironment to improve regenerative conditions; and can be functionally modified for protection, including anti-inflammatory, antioxidant/ROS-scavenging, and anti-apoptotic effects, thereby enhancing neuronal survival and repair.

## 5. Specific Applications of Hydrogels in TBI and Recent Research Advances

### 5.1. Drug Loading and Controlled-Release Systems

Drug loading and controlled-release systems play a pivotal role in the therapeutic application of hydrogels for TBI. These systems enable sustained and localized delivery of therapeutic agents, thereby attenuating neuroinflammation and oxidative stress while markedly enhancing overall efficacy. Hydrogels serve as effective carriers for a range of therapeutic agents, including dexamethasone, curcumin, and DFO. Their three-dimensional network and high water content enable stable encapsulation and gradual release directly at the injury site, thereby prolonging therapeutic action while minimizing systemic side effects [59,60].

For example, an injectable mesoporous polydopamine/dexamethasone-loaded hydrogel (MPDA@DEX@gel) achieved sustained dexamethasone release in TBI models, yielding 2–3-fold higher local drug concentrations, significant suppression of microglial activation and pro-inflammatory cytokines, and marked improvements in neurological scores relative to systemic administration [37,61]. Similarly, DFO-loaded ROS-responsive hydrogels with dynamic boronate ester networks respond to elevated post-traumatic ROS levels, enabling precise drug release, modulation of oxidative stress, reduction of iron overload-induced ferroptosis, and promotion of neuroregeneration [38].

The physicochemical properties of hydrogels, including pH- and temperature-sensitivity, further enable precise control of release kinetics. By modulating polymer concentration, crosslinking density, and nanoparticle integration, tailored release profiles can be achieved across different phases of TBI repair [62,63]. Nanoparticle–hydrogel composites have emerged as a promising strategy, offering dual-controlled release that enhances local drug stability and targeting while reducing systemic toxicity. These systems are particularly advantageous in TBI, where high local drug concentrations at the injury site are essential for effective neuroprotection [37,64].

In addition to enabling sustained and localized release, hydrogel systems dramatically improve drug penetration across the BBB compared with conventional systemic administration. Systemic drugs typically achieve only 1–5% BBB penetration, resulting in sub-therapeutic brain concentrations and substantial off-target systemic toxicity [18,65]. In contrast, injectable hydrogels enable direct and sustained release into the lesion cavity, achieving near-100% local bioavailability. For example, the MPDA@DEX@gel nanocomposite hydrogel attained 2–3-fold higher local dexamethasone concentrations than systemic injection while markedly suppressing microglial activation and pro-inflammatory cytokines in TBI models [65]. Similarly, ROS-responsive HA-PBA/PVA/DFO hydrogels delivered DFO with sustained release over 7–14 days, resulting in an approximately 50% reduction in iron overload and ROS levels without detectable systemic exposure [38]. These quantitative advantages—near-complete local bioavailability, a prolonged therapeutic window of days to weeks, and elimination of first-pass metabolism—highlight how hydrogel-based systems overcome a fundamental limitation of conventional pharmacotherapy in TBI.

### 5.2. ROS-Responsive Hydrogels for Precise Regulation of Oxidative Stress via Dynamic Boronate Ester Bonds

Following TBI, excessive ROS constitute a critical mediator of secondary neuropathology. Precise modulation of ROS levels is essential to attenuate neuronal damage and promote tissue repair. ROS-responsive hydrogels exploit redox-sensitive dynamic boronate ester bonds to dynamically modulate their network structure, thereby enabling targeted drug delivery and efficient ROS scavenging [38,66].

These hydrogels form reversible boronate ester crosslinks between HA-PBA and PVA, conferring rapid responsiveness to ROS, including hydrogen peroxide (H_2_O_2_). At elevated ROS concentrations (0.1–1 mM, characteristic of the acute TBI microenvironment), the boronate bonds undergo rapid deboronation and cleavage, resulting in network relaxation and controlled degradation. This mechanism enables automatic, on-demand release of encapsulated therapeutics in response to local oxidative stress, thereby preventing wasteful continuous release and minimizing off-target effects [38,67,68].

A representative system is the DFO-loaded HA-PBA/PVA hydrogel. In the Feeney weight-drop model of moderate TBI in Sprague–Dawley rats, this hydrogel achieved rapid in situ gelation (<5 min) upon injection into the lesion cavity. Under ROS-rich conditions, it displayed tunable degradation kinetics (complete network breakdown within 7–14 days) and sustained DFO release, resulting in an approximately 50% reduction in iron overload and lipid peroxidation within the lesion area. These effects led to significant attenuation of ROS accumulation, inhibition of ferroptosis-related neuronal damage, reduced neuroinflammation (decreased microglial activation and pro-inflammatory cytokine levels), preservation of BBB integrity, and marked improvements in motor function and neurological scores at 4 weeks post-injury, accompanied by substantially smaller contusion volumes compared with vehicle controls [38]. The system also exhibited excellent biocompatibility and injectability, rendering it highly suitable for minimally invasive clinical translation.

Compared with other ROS-sensitive chemistries, such as thioether or polysulfide linkages, boronate ester-based systems offer superior sensitivity and more tunable degradation kinetics. Boronate esters undergo rapid deboronation and bond cleavage at low H_2_O_2_ concentrations (0.1–1 mM), which are characteristic of the acute TBI microenvironment, thereby enabling on-demand drug release within hours. In contrast, thioether-based systems typically require substantially higher ROS thresholds (>10 mM) and display slower degradation over days to weeks. This superior sensitivity, combined with controllable degradation kinetics, allows boronate ester hydrogels to achieve precise temporal matching with the dynamic oxidative stress profile after TBI, thereby providing more accurate and efficient neuroprotection than alternative ROS-responsive systems [38,57,66].

In summary, dynamic boronate ester-based ROS-responsive hydrogels precisely sense and modulate the post-TBI oxidative stress microenvironment through their unique chemistry, simultaneously providing structural support and targeted pharmacotherapy. Integration of multi-stimuli responsiveness and nanotechnology is expected to further enhance their therapeutic potential for TBI and other central nervous system disorders [38,57,61].

### 5.3. Stem Cell and Exosome Delivery Systems

Recently, stem cells and their derived exosomes have emerged as promising tools in regenerative medicine for neurological injuries, including TBI. However, their standalone clinical application is severely limited by low survival rates, poor migration, and rapid clearance. Hydrogels, with their biocompatible three-dimensional porous architecture and tunable release properties, provide an ideal platform for efficient stem cell and exosome delivery [60,69,70].

Stem cell-laden hydrogels promote neural regeneration and functional recovery by enhancing cell survival and migration. In post-TBI repair, stem cells secrete neurotrophic factors, modulate the immune microenvironment, and activate endogenous neural stem cells. Encapsulating stem cells within hydrogels provides a protective three-dimensional ECM-mimetic scaffold that shields cells from the hostile inflammatory milieu while promoting adhesion, proliferation, and migration. For example, collagen–fibrin hydrogels significantly improved neural stem cell survival and differentiation in TBI models, resulting in enhanced cortical tissue reconstruction, reduced glial scarring, and improved cognitive and behavioral function [71]. Similarly, silk fibroin (SF)-based hydrogels encapsulating human mesenchymal stem cells (hMSCs) significantly reduced neuronal death in the injured hippocampus, decreased lesion area, and improved neurological functional recovery in a rat brain injury model [72].

Exosome-loaded hydrogels enable stable and sustained release while modulating the immune microenvironment and activating endogenous neural stem cells. GelMA hydrogels incorporating stem cell-derived exosomes have been shown to promote neurogenesis, suppress neuroinflammation, and enhance angiogenesis in TBI models. In one study, a hyaluronan–collagen hydrogel integrating bone marrow mesenchymal stem cell-derived exosomes (DHC-BME) promoted angiogenesis and neurogenesis, enhanced structural remodeling and neurological recovery after TBI, and may also help mitigate adverse astrocytic responses in the lesion microenvironment [69]. Another platform, bio-orthogonal hydrogel–extracellular vesicles (BIOGEL-EVs), further demonstrated enhanced cortical reconstruction, hippocampal neurogenesis, remyelination, and suppression of neuroinflammation, resulting in substantial improvements in sensorimotor and cognitive function [73]. These exosome–hydrogel systems preserve exosome integrity and bioactivity while overcoming rapid in vivo clearance, thereby offering a cell-free therapeutic strategy with high translational potential.

In summary, the integration of stem cells and exosomes with hydrogels offers a multifunctional strategy that capitalizes on the three-dimensional scaffolding and sustained-release properties of hydrogels to enhance local retention, cell viability, and bioactivity, thereby promoting robust neural repair and functional recovery after TBI [71,74,75]. Future optimization of hydrogel biomechanics and exosome–hydrogel interactions is expected to further accelerate clinical translation.

### 5.4. Promoting Angiogenesis and Neural Network Reconstruction

Tissue repair and functional recovery after TBI depend critically on local angiogenesis and neural network reconstruction. Hydrogels, serving as three-dimensional scaffolds that mimic the native ECM, act as ideal carriers for promoting angiogenesis and neuroregeneration owing to their excellent biocompatibility and tunable physicochemical properties. Recent studies on SAP and conductive hydrogels have demonstrated considerable potential in enhancing local angiogenesis and restoring the BBB [75,76,77].

SAP hydrogels closely mimic the nanofibrillar architecture of the ECM and enable controlled release of growth factors or EVs. For example, a neuroprotective peptide hydrogel (PANAP), when injected into the lesion cavity of Wistar rats after TBI, rapidly formed a nanofibrous network that scavenged ROS and RNS, reduced oxidative stress and neuroinflammation, decreased infarct volume, protected and restored the BBB, and promoted neurogenesis. At 5 days post-injury, treated animals exhibited accelerated sensorimotor and cognitive recovery compared with controls [78]. Similarly, the SFNV sulfo-functionalized self-assembling peptide hydrogel significantly promoted neurite outgrowth, increased the expression of neuroregeneration-related markers, counteracted anti-nerve growth factor (NGF)-induced neurotoxicity, and was associated with histological signs of repair in the hippocampal dentate gyrus [79].

Conductive hydrogels impart electrical conductivity that mimics endogenous bioelectric signaling in the brain, thereby promoting neural cell growth and network reconstruction. In one study, carbon nanotube- or gold nanoparticle-modified conductive hydrogels enhanced neuronal regeneration and functional recovery in brain injury models by modulating the local electrical microenvironment and accelerating angiogenesis and BBB repair [80]. Functionalized glycosaminoglycan (GAG) hydrogels further enhance synaptic formation and axonal regeneration by providing sustained release of neurotrophic factors and abundant adhesion sites. Dual-layer GAG-GelMA hydrogels, for instance, promoted angiogenesis and NSCs activation while synergistically facilitating neurovascular network reconstruction [81].

In summary, SAP and conductive hydrogels robustly support post-traumatic microenvironment remodeling by promoting local angiogenesis and BBB restoration, whereas functionalized GAG hydrogels enhance neuronal synaptogenesis and axonal regeneration. These platforms transcend passive scaffolding to actively orchestrate neurovascular repair, thereby providing effective strategies for comprehensive TBI treatment [78,80,82,83].

### 5.5. Anti-Inflammatory and Neuroprotective Mechanisms

In the pathophysiology of TBI, inflammation and neuronal death are major drivers that exacerbate secondary injury. As ideal local drug delivery platforms, hydrogels enable precise modulation of the inflammatory microenvironment and confer robust neuroprotection [37,57,64].

Hydrogel-mediated local delivery plays a critical role in regulating microglial polarization. Following TBI, microglia predominantly polarize toward the pro-inflammatory M1 phenotype, releasing excessive mediators that exacerbate neural damage. For example, the GelMA/CSMA composite hydrogel loaded with erythropoietin (EPO) and interleukin-4 (IL-4), termed GC/I/E, enabled rapid EPO release and sustained IL-4 delivery, effectively shifting microglia toward the anti-inflammatory M2 phenotype. This system reduced brain edema and Nissl body loss, suppressed astrocyte and microglial activation, decreased pro-inflammatory cytokine levels, promoted angiogenesis and synaptic regeneration, and significantly improved neurological scores and behavioral outcomes [57].

Antioxidant-incorporated hydrogels effectively mitigate iron overload-associated ferroptosis. For instance, a post-trauma microenvironment-responsive ROS-depleting TM/PC hydrogel containing PPS120 and curcumin (Cur) was developed for TBI treatment. This hydrogel effectively depleted ROS, reduced oxidative stress and neuroinflammation, and promoted neuroregeneration and functional recovery in ICR mouse TBI model [66].

Multifunctional hydrogels further integrate neuroprotection through synergistic mechanisms. For example, the Gel/PDA-AMSN-D system reduced lesion volume, protected BBB integrity, and accelerated neurological functional recovery through combined antioxidant and neuroprotective effects [84].

In summary, hydrogels exhibit substantial potential in anti-inflammatory and neuroprotective strategies for TBI by enabling precise drug delivery to regulate microglial polarization, scavenging ROS to attenuate iron overload-induced ferroptosis, and integrating multifunctional synergy for comprehensive neuroprotection. These attributes provide a robust mechanistic foundation for the clinical translation of hydrogel-based TBI therapies [48,57,84,85].

### 5.6. Comparative Analysis of Hydrogel Platforms

Direct comparisons among different hydrogel platforms highlight platform-specific strengths and limitations rather than universal superiority. ROS-responsive hydrogels (e.g., HA-PBA/PVA/DFO and TM/PC) excel in acute oxidative stress control, achieving rapid network degradation and an approximately 50% reduction in ROS levels within hours to days, yet suffer from faster clearance and limited long-term structural support [38,66]. In contrast, ECM-derived hydrogels offer superior sustained tissue integration, angiogenesis, and neurogenesis over 4–8 weeks [39,57], yet often require more invasive delivery and exhibit slower initial anti-inflammatory effects. Stem cell- and exosome-loaded systems provide excellent immunomodulation and endogenous NSCs activation, significantly improving modified Neurological Severity Scores (mNSS) [61,69], but face challenges related to cell/exosome viability and batch-to-batch consistency. SAP hydrogels exhibit excellent nanofibrillar ECM mimicry and neuroprotection [78]. Overall, no single platform is optimal for all TBI phases; hybrid multicomponent systems that combine ECM mimicry with stimuli-responsiveness represent the most promising avenue for precision therapy [53,68].

## 6. Challenges and Future Development Directions in Hydrogel Applications

### 6.1. Current Application Limitations

Hydrogels, as emerging biomaterials for TBI therapy, hold considerable promise owing to their high water content, excellent biocompatibility, and ability to mimic the native ECM. Nevertheless, their current applications face several practical limitations, including a mismatch between biodegradation rates and the dynamic timelines of tissue repair, difficulties in maintaining drug and cell carrier stability with controlled release, and concerns regarding in vivo immune responses and long-term biocompatibility [7,53,86].

Biodegradation rates frequently fail to align with the dynamic requirements of post-TBI repair, which encompasses neuronal regeneration, angiogenesis, and inflammation modulation. Ideal hydrogels should degrade in synchrony with tissue remodeling, thereby providing sustained structural support without premature breakdown or long-term persistence that could impede natural healing. Hydrogels derived from natural or synthetic polymers exhibit substantial variation in degradation kinetics and mechanical properties, rendering precise control through chemical modification and crosslinking technically challenging [7,75].

Drug and cell carrier stability together with controlled release remain major challenges in the complex brain microenvironment, where inflammation and enzymatic activity can readily compromise therapeutic integrity and cell viability [53,62]. In vivo immune responses and biocompatibility also warrant careful consideration, as implanted hydrogels may elicit foreign-body reactions or chronic inflammation, particularly those based on synthetic polymers [53,55,79].

This review is inherently limited by its reliance on predominantly preclinical rodent data, most of which have not yet been independently replicated at scale. Publication bias favoring positive outcomes is likely present in the hydrogel field, with negative or null-result studies potentially underrepresented. Moreover, heterogeneity in TBI models, follow-up durations, and outcome measures further restricts direct synthesis of findings. In accordance with the predefined inclusion and exclusion criteria outlined in Section 2—which focused exclusively on hydrogel applications (as drug delivery platforms, scaffolds, stem cell/exosome carriers, or stimuli-responsive systems) in preclinical TBI models or clinically relevant in vitro studies—emerging clinical dural-sealant applications and non-hydrogel nanocarriers were not covered in depth. These limitations should be taken into account when interpreting the synthesized evidence.

### 6.2. Future Research Trends and Technological Innovations

With the growing clinical demand for TBI treatment and continued advances in hydrogel technology, future innovations are expected to focus on multifunctional stimuli-responsive hydrogels, nanotechnology integration, biomimetic 3D scaffolds that emulate the brain microenvironment, accelerated clinical translation, and synergistic combinations with gene editing and stem cell therapies.

Multifunctional stimuli-responsive hydrogels capable of precise spatiotemporal control represent a major research direction. These hydrogels undergo reversible or irreversible physicochemical changes in response to endogenous cues (e.g., pH, enzymes, or redox conditions), thereby enabling controlled release and targeted delivery that enhance therapeutic efficacy. For instance, stimuli-responsive hydrogels have been shown to markedly improve neurological recovery in TBI through neuroregeneration, anti-inflammatory, and antioxidant effects [8]. Furthermore, dynamic hydrogels with tunable mechanical properties and reversible crosslinking closely mimic native brain tissue, facilitating both in vitro disease modeling and advanced TBI therapy [47]. Such designs are poised to advance TBI treatment toward greater precision and personalization.

Integration of nanotechnology with hydrogels markedly enhances delivery efficiency and targeting precision. Nanoparticles can effectively traverse the BBB; when combined with the localized and sustained release provided by hydrogels, they enable highly efficient drug delivery. For example, conductive microporous hydrogels incorporating gold nanowires promote angiogenesis, neuronal survival, and motor recovery through electromagnetic stimulation [75]. Concurrently, antioxidant-encapsulating nanoparticles (e.g., flavonoids) can be engineered to release in response to injury-specific cues, thereby reducing oxidative stress and accelerating tissue repair [66]. Overall, nanotechnology integration substantially augments both the functionality and therapeutic efficacy of hydrogels.

Biomimetic 3D hydrogel scaffolds that emulate the brain microenvironment and facilitate neural network reconstruction represent a central focus in regenerative medicine. ECM-based functionalized hydrogels replicate the biochemical and mechanical cues of brain tissue, thereby supporting the adhesion, migration, differentiation, and regeneration of NSCs [54,55]. For example, neurotrophic factor-loaded peptide/hyaluronic acid composite hydrogels have been shown to promote neuronal growth and restore neurological function [60,79]. In addition, the tunable porosity of 3D scaffolds enhances angiogenesis and intercellular signaling, making them particularly suitable for TBI repair.

Enhanced clinical translation remains a critical priority for hydrogel-based TBI therapies. Although these materials have shown considerable promise in preclinical animal models, systematic evaluations of safety and long-term efficacy remain insufficient. Comprehensive assessments of biocompatibility, degradation byproducts, immune responses, and long-term effects on brain function are therefore essential [7]. Moreover, future clinical trials should prioritize optimal therapeutic windows, dosing regimens, and combination strategies to facilitate successful clinical implementation.

The integration of gene editing and stem cell therapy with hydrogels offers a promising pathway toward personalized TBI treatment strategies. Acting as three-dimensional carriers, hydrogels protect stem cells from immune rejection while promoting their survival and differentiation through sustained neurotrophic factor release [72]. Incorporation of CRISPR-based gene editing enables precise modulation of both cellular behavior and the local microenvironment according to individual genetic and pathological profiles, thereby facilitating customized repair. Furthermore, hydrogel-mediated delivery of stem cell-derived exosomes can reduce immune responses and significantly enhance neuroregenerative efficacy [68,69,73].

In summary, future research on hydrogels for TBI should prioritize the development of multifunctional stimuli-responsive composites. The integration of nanotechnology and biomanufacturing is expected to generate highly biomimetic 3D microenvironments. Accelerated clinical translation, together with the incorporation of gene editing and stem cell therapies, will pave the way for precise, personalized TBI repair strategies. Ultimately, these innovations hold the potential to substantially improve neurological recovery and quality of life for patients with TBI.

### 6.3. Translational Challenges and Future Strategies

Although preclinical studies in rodent TBI models have demonstrated promising neuroprotective and regenerative effects of hydrogels, none have yet advanced to clinical use for intraparenchymal brain injury. This translational gap arises from several critical barriers. First, substantial neuroanatomical differences exist between rodents and humans: the rodent brain is lissencephalic and substantially smaller, with a distinct white-matter architecture compared with the gyrencephalic human brain. These differences result in discrepancies in lesion volume scaling, secondary injury propagation, and functional outcome assessment [7,26]. Second, hydrogel scalability remains a major challenge. Most formulations optimized for microliter-scale rodent injections are difficult to manufacture at the larger volumes required for human TBI cavities while preserving uniform mechanical properties and sterility [8]. Third, key manufacturing and regulatory hurdles—including terminal sterilization, long-term shelf-life stability under physiological conditions, and compliance with Good Manufacturing Practice (GMP) standards—have not been systematically addressed in the current literature [60]. Finally, the U.S. Food and Drug Administration (FDA) classifies most implantable hydrogels as Class III devices, which require rigorous safety and efficacy data from large-animal models (e.g., porcine or nonhuman primate) before human trials can commence.

All hydrogel platforms discussed in this review remain at the preclinical stage and have been evaluated exclusively in rodent TBI models. In terms of translational potential, the biomaterial-based strategies reviewed here can be broadly grouped into three categories. First, platforms combining an already clinically recognized drug with localized delivery appear to be the most immediately translatable, as exemplified by MPDA@DEX@gel. The key advantage of this class lies in the fact that the therapeutic cargo already has a well-defined pharmacological background, while local administration directly addresses major limitations of systemic treatment, including poor blood–brain barrier penetration and off-target adverse effects. Accordingly, the principal translational challenges are more likely to center on device formulation, release control, and surgical standardization, rather than on de novo therapeutic validation [37]. Second, hydrogel–extracellular vesicle systems and multifunctional bioactive platforms generally provide the most comprehensive preclinical efficacy packages, as illustrated by BIOGEL + DFO-EVs, GC/I/E, and SDF@HA/BM. These studies typically integrate behavioral recovery, histological repair, and mechanistic readouts, thereby offering a stronger biological proof-of-concept than simpler formulations. However, this greater mechanistic and therapeutic sophistication is accompanied by increased translational complexity, particularly with respect to scalable manufacturing, quality control, batch-to-batch consistency, and regulatory classification. Thus, these platforms may be the most compelling scientifically, but not necessarily the fastest to reach clinical translation [54,57,73]. Third, cell-based, genetically modified cell-based, and cell-composite approaches may offer the greatest regenerative potential, but they also face the highest translational barriers. Representative examples include mNSC spheroid sheet, BDNF-hMSC, and HT+NGF+BMSC. For these strategies, the major bottleneck is often not proof of efficacy per se, but rather the development of a clinically deployable product with stable manufacturing, reproducible potency, long-term safety, acceptable immunological compatibility, and feasible cost and supply-chain management. As a result, despite promising biological effects, their overall translational readiness remains comparatively lower at the current preclinical stage [60,61,72]. Overall, these studies suggest that the shortest path to clinical translation is most likely to come from localized delivery systems built around known therapeutic agents, whereas multifunctional EV-based biomaterials may represent the most biologically comprehensive but developmentally demanding class, and cell-centered strategies, although highly promising, still require the most substantial advances in manufacturing and safety standardization before clinical implementation can be realistically pursued.

To bridge this gap, future efforts should prioritize: (1) validation in large-animal TBI models that more closely recapitulate human brain anatomy and injury scale; (2) development of shear-thinning, ready-to-use injectable hydrogels with extended shelf-life and terminal sterilization compatibility; and (3) early engagement with regulatory agencies to design adaptive clinical trials incorporating imaging-guided delivery and long-term functional endpoints. Addressing these concrete challenges will be essential for translating hydrogel-based therapies from bench to bedside in TBI treatment.

## 7. Conclusions

Hydrogels have achieved substantial progress as emerging biomaterials for TBI therapy, demonstrating significant advances in neuroprotection, neuroregeneration, inflammation modulation, and angiogenesis. These achievements stem from their unique biophysical properties and multifunctionality (as summarized in Appendix A). Collectively, they have deepened our understanding of TBI pathophysiology and provided innovative platforms for precision medicine. Recent innovations in drug loading, stem cell/exosome delivery, and stimuli-responsive mechanisms have successfully overcome many limitations of conventional therapies, laying a solid foundation for personalized and multitargeted treatment strategies.

Nevertheless, significant challenges remain. Hydrogel biodegradability and biocompatibility directly influence in vivo persistence and safety, while immune responses may trigger adverse reactions that limit clinical adoption. Translating laboratory findings to clinical practice is further complicated by complexities in material design, scalable manufacturing, and trial design. Future progress will require deeper interdisciplinary integration between materials science and biomedicine, along with systematic investigations of hydrogel–brain interactions. Such efforts will enable optimization of both physicochemical and biological properties, while enhancing stimuli-responsiveness and targeted delivery. Although the academic community widely recognizes the transformative potential of hydrogels for TBI, ongoing debates persist regarding optimal pathways and long-term safety. Some studies emphasize improved biodegradation control through refined formulations and crosslinking strategies, whereas others advocate for multifunctional composites and nanotechnology to address complex pathological mechanisms. Ultimately, multidisciplinary collaboration will be essential to bridge basic and clinical research, evaluate diverse approaches through systematic, evidence-based comparisons, and accelerate the identification of truly transformative solutions.

Hydrogels hold immense potential as a transformative approach to TBI treatment. Continued breakthroughs in materials science, combined with deeper insights into neural repair from biomedicine, will evolve hydrogels from simple carriers into intelligent, multifunctional platforms. Standardized clinical trials and long-term follow-up studies will generate robust safety and efficacy data, thereby accelerating their clinical adoption. Ultimately, these advances are expected to markedly improve neurological recovery and quality of life for TBI patients while substantially alleviating the associated socioeconomic burden.

In summary, hydrogel technology represents a promising and innovative direction for TBI treatment. Although grounded in robust scientific evidence, it still faces several practical challenges. Sustained mechanistic exploration, multidisciplinary collaboration, and rigorous clinical validation will be essential to fully realize its potential and establish hydrogels as a cornerstone of TBI therapy. The ongoing convergence of materials science and neurology will be pivotal in achieving precise, personalized treatment and rehabilitation strategies for TBI.

## Data Availability

No datasets were generated or analysed during the current study.

## Appendix A

**Table A1:** Therapeutic mechanisms of hydrogels for TBI.

Hydrogels	Models	Outcome	Functional Endpoint	Follow-Up Duration	Effect Magnitude	References
HA/Gel/SAB/VEGF hydrogel	In vitro and C57BL/6 mice	• Fills lesion cavity and supports tissue ingrowth • Promotes angiogenesis and neurogenesis • Reduces cavity volume and tissue loss • Improves neurological and cognitive recovery	Brain cavity/defect volume assessed by gross observation and H&E staining	28 days; assessed at days 0, 14, and 28 after intracerebral hydrogel injection	HA/Gel/SAB/VEGF vs. Control: cavity volume ~0.15 vs. 0.15 mm^3^ (day 0), 0.12 vs. 0.18 mm^3^ (day 14), and 0.05 vs. 0.19 mm^3^ (day 28); absolute reduction at day 28 ~0.14 mm^3^	[39]
GC/I/E hydrogel	In vitro and C57BL/6J mice	• Reduces brain edema and Nissl body damage • Suppresses excessive astrocyte and microglia activation • Decreases pro-inflammatory cytokines • Promotes angiogenesis, neurogenesis and synaptic regeneration • Improves neurological scores and behavioral outcomes	mNSS, NOR discrimination index, and MWM outcomes	28 days; mNSS: days 1, 3, 7, 14, 28; MWM: days 15–20; NOR: day 27/28	GC/I/E vs. Control: mNSS ~2.7 vs. 2.8 (day 1), 4.2 vs. 5.3 (day 3), 3.3 vs. 5.8 (day 7), 1.3 vs. 2.0 (day 14), 1.0 vs. 2.0 (day 28); NOR ~64% vs. 51%; MWM escape latency ~71 vs. 64 s (day 15) and 22 vs. 68 s (day 19); platform crossings ~2.1 vs. 1.1; time in target zone ~24% vs. 17%	[57]
SDF@HA/BM hydrogel	In vitro and C57BL/6J mice	• Counteracts TBI-induced acute immunosuppression and restores immune cell function • Normalizes inflammatory cytokine levels • Promotes their neuronal differentiation and neuroprotection • Facilitates brain tissue remodeling, reduces demyelination and promotes remyelination • Improves motor and spatial learning/memory and alleviates depression- and anxiety-like behaviors	mNSS, balance beam score, tail suspension struggle time, Morris water maze outcomes, and open field outcomes	56 days; mNSS, balance beam, tail suspension: days 3, 7, 14, 21; MWM: training on days 23–27 with spatial exploration on day 28; open field: day 32	SDF@HA/BM vs. TBI: mNSS 1 ± 1 vs. 3 ± 1 (day 21); tail suspension struggle time 278 vs. 211 s (day 21); MWM escape latency 26.2 vs. 43.5 s (day 27); latency to target 15.1 vs. 41.8 s (probe test); open field total distance 1819 vs. 1342 cm and central zone entries 19 vs. 12; balance beam score was also improved at day 21	[54]
(CS-A)CP hydrogel	In vitro and SD rats	• Induces NSC differentiation toward neurons with long neurites and increased spontaneous Ca2+ activity • Prevents neuronal and axonal loss and reduces brain atrophy • Accelerates recovery of skilled forelimb reach-to-grasp function	Skilled reach task (SRT) outcomes	5 weeks after sTBI; behavioral assessment performed weekly at weeks 1, 2, 3, 4, and 5 post-injury	(CS-A)CP vs. CCI-SA: animals showed significantly accelerated recovery of reach-to-grasp function over 5 weeks; SRT performance was comparable to Sham by week 1, whereas CCI-SA animals had persistent deficits through week 5. Hydrogel-implanted animals also showed significantly improved retrieval efficiency, reduced assay duration, and higher success rates than sTBI controls across the 5-week follow-up.	[55]
HT/HGA hydrogel	In vitro and C57BL/6 mouse	• Efficiently scavenges ROS in the injured brain • Attenuates oxidative stress and neuroinflammation • Protects blood–brain barrier integrity and reduces brain edema • Decreases tissue loss and neuronal damage • Improves motor and cognitive function	mNSS, sucrose preference test (SPT), and Morris water maze (MWM) outcomes	21 days; mNSS: days 1, 3, 7, 14, 21; MWM and SPT: days 16–21	HT/HGA vs. NS: mNSS was significantly lower at day 7, 14, and 21; SPT sucrose preference index was significantly increased at day 21; in MWM, escape latency was reduced, time in target zone was increased, and platform crossings were increased versus NS, with HT/HGA also outperforming HT in time in target zone and platform crossings	[48]
HA-PBA/PVA/DFO hydrogels	In vitro and SD rats	• Lowers iron overload and lipid peroxidation in the lesion area • Inhibits ROS accumulation and ferroptosis-related damage • Reduces neuroinflammation and neuronal loss • Improves motor function and neurological behavior	mNSS, beam-walking test, hang wire test, and open-field test (OFT) outcomes	14 days; mNSS, beam-walking test, and hang wire test: days 0, 1, 3, 7, 14; OFT: day 14	HA-PBA/PVA/DFO vs. TBI: mNSS was significantly improved at days 1, 3, 7, and 14; hang wire test score was significantly higher at days 1, 3, 7, and 14; in the OFT, rats showed longer total movement distance, higher mean speed, longer central-area time, and higher central-area entry frequency than the TBI group.	[38]
TM/PC hydrogel	In vitro and ICR mice	• Significantly decreases ROS levels and protects the BBB • Reduces brain edema and tissue damage • Suppresses astrocyte/microglia activation and inflammatory cytokine expression • Improves motor function and spatial memory	mNSS, wire hanging test, and Morris water maze (MWM) outcomes	21 days; mNSS: days 0, 1, 3, 7, 14; wire hanging test: days 3, 7, 14, 21; MWM: training during days 14–20 with probe test on day 21	TM/PC vs. TBI: mNSS was significantly lower by day 14 in both WDI and PBI models; wire hanging latency was significantly longer at days 3, 7, 14, and 21; in MWM, escape latency was significantly shorter on training days 4–7, and time in target quadrant on the probe trial (day 21) was significantly increased, while swimming speed showed no significant between-group difference.	[66]
Collagen- fibrin hydrogel	In vitro and C57BL/6J mice	• Promotes cortical tissue reconstruction and angiogenesis • Reduces degenerating neurons and glial reaction • Improves cognitive and behavioral function	Novel object recognition (NOR) preference index and Y-maze alternation	28 days after transplantation following TBI; NOR: 7, 14, and 28 days post-transplantation (DPT); Y-maze: 7 DPT	mNSC spheroid sheet vs. mTBI: NOR preference index (A1:C1) ~61% vs. 49% (7 DPT), ~61% vs. 44% (14 DPT), and ~56% vs. 44% (28 DPT); Y-maze alternation at 7 DPT ~60% vs. 48%. Compared with mNSC spheroids alone, the mNSC spheroid sheet also showed higher NOR preference index at 14 DPT (~61% vs. 50%) and 28 DPT (~56% vs. 46%), and higher Y-maze alternation at 7 DPT (~60% vs. 54%)	[61]
GelGMA/SF hydrogel	In vitro and SD rats	• Reduces neuronal death in the injured hippocampus • Decreases lesion/cavity volume • Improves neurological recovery	mNSS and MRI lesion area; supportive histological endpoints included FJB-positive neuronal degeneration, NeuN-positive neuronal survival, and MAP2 intensity in the hippocampus	21 days after trans-septal transplantation; mNSS: 1 h, 1, 2, 3, 4, 5, 6, and 7 days post-TBI; MRI: 3, 7, 14, and 21 days post-TBI; acute histology: 3 days; chronic histology/Western blot: 7 days	BDNF-hMSC vs. vehicle: mNSS ~14 vs. 14 (1 h), 10 vs. 12 (day 1), 8 vs. 10 (day 2), 6 vs. 9 (day 3), 5 vs. 8 (day 4), 4 vs. 7 (day 5), 3 vs. 6 (day 6), and 2 vs. 5 (day 7); ΔmNSS was also higher in the BDNF-hMSC group across 1h–1d through 1h–7d intervals. MRI lesion area was significantly reduced at 14 and 21 days in the BDNF-hMSC group. At 3 days, hippocampal neuronal degeneration was markedly reduced, with FJB-positive cells in CA1, GCL, and hilus decreased to about one-third to one-fourth of vehicle, while MAP2 intensity, NeuN-positive cell counts, and hippocampal BDNF expression were significantly increased.	[72]
HT/BMSC/NGF hydrogel	In vitro and C57BL/6 mice	• Modulates the inflammatory microenvironment • Reduces inflammation and neuronal death in the injury area and decreases lesion volume • Significantly improves mNSS neurological scores and learning/memory in the Morris water maze • Accelerates functional recovery after TBI	mNSS and Morris water maze (MWM) outcomes	28 days after implantation; mNSS: days 1, 3, 7, 14, 21, 28; MWM: days 23–28	HT+NGF+BMSC vs. NS: mNSS ~8.9 vs. 9.1 (day 1), 7.3 vs. 7.8 (day 3), 5.5 vs. 6.5 (day 7), 3.2 vs. 5.4 (day 14), 2.1 vs. 4.7 (day 21), and 1.8 vs. 4.3 (day 28); in MWM, escape latency fell to ~8 s vs. 35 s by the final training day, platform crossings increased to ~2.0 vs. 0.2, and time in target quadrant increased to ~48% vs. 22%	[60]
DHC-BME hydrogel	In vitro and SD rats	• Promotes angiogenesis and neuroregeneration in the lesion area • Reduces glial scar formation and neuroinflammation • Enhances neurological score and motor/cognitive recovery	mNSS and Morris water maze (MWM) outcomes	28 days after TBI; mNSS: days 1, 3, 7, 14, 21, 28; MWM: days 21–26 with probe assessment at 28 dpi	DHC-BME vs. TBI: mNSS ~10 vs. 11 (day 1), 8 vs. 9.5 (day 3), 6.5 vs. 8 (day 7), 4.5 vs. 7 (day 14), 3.5 vs. 6 (day 21), 2.5 vs. 5 (day 28); MWM escape latency ~49 vs. 52 s (day 21), 31 vs. 44 s (day 22), 23 vs. 40 s (day 23), 18 vs. 34 s (day 24), 15 vs. 30 s (day 25), 12 vs. 26 s (day 26); time in target quadrant ~35% vs. 20% and site crossings ~6 vs. 2 at 28 dpi	[69]
BIOGEL-EV hydrogel	In vitro and SD rats	• Enhances brain tissue regeneration and cortical reconstruction • Promotes hippocampal neurogenesis and remyelination • Suppresses neuroinflammation • Improves sensorimotor and cognitive recovery	mNSS and Rotarod latency	28 days after TBI; mNSS: pre, 7, 14, 21, 28 days; Rotarod: pre, 7, 14, 21, 28 days	BIOGEL + DFO EV vs. TBI: mNSS ~0 vs. 0 (pre), 7.0 vs. 10.8 (day 7), 6.0 vs. 9.9 (day 14), 5.1 vs. 9.2 (day 21), and 4.5 vs. 8.8 (day 28); text-reported day 28 mNSS 4.53 ± 0.36 vs. 8.77 ± 0.35. Rotarod latency in the treatment group recovered to ~10 s (day 7), ~130 s (day 14), ~145 s (day 21), and ~152 s (day 28), approaching sham performance (~200 s at day 28)	[73]
MPDA@DEX@gel hydrogel	In vitro and C57BL/6 mice	• Effectively suppresses neuroinflammation and microglia/astrocyte activation • Reduces brain edema and tissue damage • Promotes neuronal survival • Improves neurological scores and behavioral recovery	mNSS and Morris water maze (MWM) outcomes	14 days after TBI; mNSS: days 1, 3, 7, 14; MWM: training on days 8–12 with probe trial on day 13	MPDA@DEX@gel vs. CCI: mNSS ~6.0 vs. 8.0 (day 3) and ~4.5 vs. 7.0 (day 7); MWM escape latency was consistently reduced across training days 1–5; in the probe trial, time in goal quadrant and distance in goal quadrant were both significantly increased versus CCI. Histologically, NeuN-positive cells were increased in both cortex and hippocampus at day 7	[37]
PANAP hydrogel	In vitro and Wistar rats	• Scavenging ROS/RNS and reducing oxidative stress and neuroinflammation • Decreases infarct volume and protects/rebuilds BBB • Reduces structural brain damage and neuronal apoptosis • Promotes neurogenesis and neural regeneration • Accelerates sensorimotor and cognitive functional recovery	Morris water maze (MWM) outcomes	7 days after CBI; treatment on day 1 and MWM/neurobehavioral assessment during days 1–7	Gel/PDA-AMSN-D vs. Injury: during the 5-day learning phase, searching time decreased to ~25 s vs. 45 s on the final day; distance to platform decreased to ~1200 cm vs. 2200 cm; in the probe trial, time in target quadrant increased to ~35 s vs. 15 s, time spent at platform increased to ~5 s vs. 2 s, and platform crossings increased to ~4 vs. 2	[78]
SFNV hydrogel	In vitro and C57BL/6 mice	• Promotes neurite outgrowth and increases expression of neuroregeneration markers • Provides significant neuroprotection and counteracts anti-NGF-induced neurotoxicity • Accelerates behavioral and histological recovery and supports neurorepair in the dentate gyrus region	Cortical tissue loss/repair assessed by cresyl violet staining, and GFAP-positive reactive astrocyte expression in the hippocampal dentate gyrus (DG) and cortex	7 days after sham injury; histological assessment performed at days 5 and 7	SFNV vs. sham injury: by day 5 and day 7, no visible cortical tissue loss/injury mark was observed in the SFNV-treated brains, whereas untreated sham-injured brains still showed a distinct cortical lesion; GFAP expression in the DG was significantly increased in the SFNV group versus sham (approximately 90 vs. 55 a.u.)	[79]
